# Legitimate Pharmacy

**Published:** 1866-07

**Authors:** 


Legitimate Pharmacy.—We are invited to call the attention of physicians and others to the notice in our advertisement sheet of Walker, Gleason & Co., who propose to deal only in legitimate medicine, and thus to merit the confidence and support of the profession. They have opened a new drug store, in convenient and attractive location, and will doubtless receive the favor they merit.

